# Genome Sequence of Ruloma Virus, a Novel Paramyxovirus Clustering Basally to Members of the Genus *Jeilongvirus*

**DOI:** 10.1128/MRA.00325-21

**Published:** 2021-05-06

**Authors:** Bert Vanmechelen, Sien Meurs, Zafeiro Zisi, Joëlle Goüy de Bellocq, Magda Bletsa, Philippe Lemey, Piet Maes

**Affiliations:** a KU Leuven–University of Leuven, Department of Microbiology, Immunology and Transplantation, Laboratory of Clinical and Epidemiological Virology, Rega Institute for Medical Research, Leuven, Belgium; b The Czech Academy of Sciences, Institute of Vertebrate Biology, Brno, Czech Republic; DOE Joint Genome Institute

## Abstract

We report here the complete genome sequence of ruloma virus, a novel paramyxovirus detected in a Machangu’s brush-furred rat from Tanzania. Ruloma virus has the longest orthoparamyxovirus genome reported to date and forms a sister clade to all currently known members of the genus *Jeilongvirus*.

## ANNOUNCEMENT

The recently established genus *Jeilongvirus* within the subfamily *Orthoparamyxovirinae* contains seven recognized species, all identified in rodents and bats (1). However, several more putative species have been reported, also in other mammals, indicating that they might be more prevalent and have a wider host range than previously assumed (2–4). Jeilongviruses are set apart from other paramyxoviruses by the presence of one or two additional genes (compared to the traditional N-P/V/C-M-F-G-L genome organization), encoding transmembrane proteins, between the F and G genes. Additionally, members of the rodent subclade of the genus *Jeilongvirus* are characterized by the long but variable length of their G genes and their overall large genome size (5).

**FIG 1:**
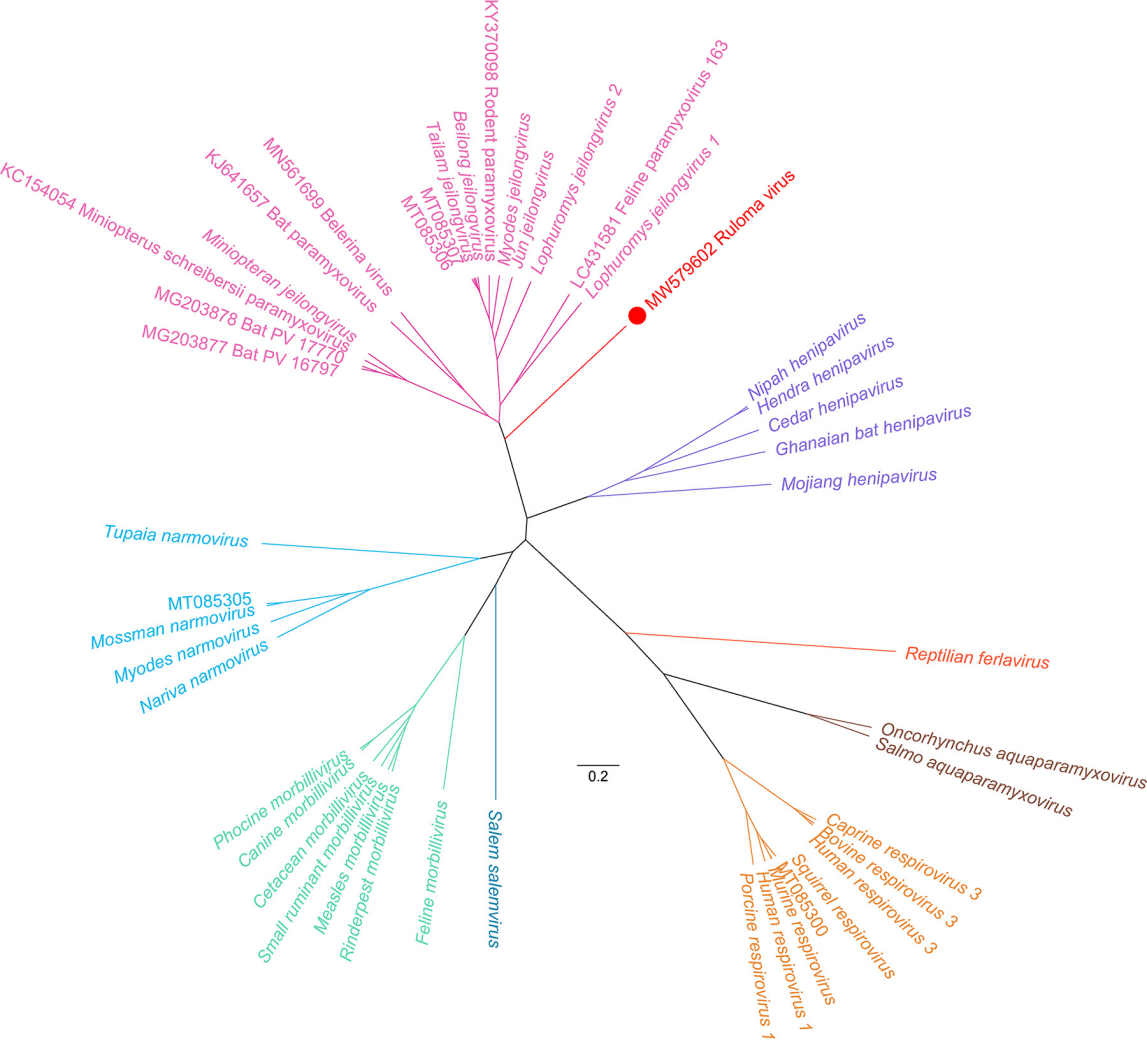
Phylogenetic tree based on the deduced amino acid sequence of the six major open reading frames (N-P-M-F-G-L) of all (putative) member species of the subfamily *Orthoparamyxovirinae* for which (near-) complete genome sequences are available. Ruloma virus is marked with a red circle. Proteins were aligned separately using MAFFT v7.310 (10). The resulting alignments were concatenated and trimmed using TrimAl v1.4.rev15 (gappyout setting) (11). Following model selection using IQ-TREE, BEAST v1.10.4 was used to infer phylogenetic trees, employing a Yule process for tree priors and LG+G4+I as an amino acid substitution model (12–15). TreeAnnotator v1.10.4 was used to summarize a maximum clade credibility tree, which was visualized using FigTree v1.4.3. Sequences are colored according to the genus in which they cluster. For recognized species, the accession number as provided in the ICTV master species list was used (16). For putative species, the accession number is shown in the tree. All nodes had a posterior support of 1, with the exception of the node between MT085307 and Tailam virus, which had a support value of 0.74.

We performed next-generation sequencing on cDNA from kidney and spleen tissue of a Machangu’s brush-furred rat (Lophuromys machangui) caught by snap-trapping in Tanzania (9°02′26.7″S, 33°34′23.5″E) in 2013 (see also reference 6). Prior to Illumina sequencing, the tissue was subjected to total RNA extraction using the RNeasy minikit (Qiagen), with additional freeze-thaw steps before and after tissue homogenization and with on-column DNase treatment (see reference 6 for a more detailed description). The RNA Quantifluor system (Promega) and a Bioanalyzer 2100 device, using an Agilent RNA 6000 Nano chip (Agilent Technologies), were used for quantitation and quality assessment. Next, the Ribo-Zero rRNA removal kit (Illumina) was used to deplete rRNA, and sequencing libraries were prepared with the NEXTflex rapid Illumina directional RNA-Seq library prep kit (PerkinElmer). Paired-end sequencing (2 × 150 bp) was performed on an Illumina NextSeq 500 instrument (Illumina) at Viroscan3D (Lyon, France). Following adapter trimming using Trimmomatic v0.36, CLC Genomics Workbench v10.0.1 was used for *de novo* assembly. Paramyxoviral contigs were identified using a tBLASTx v2.5.0+ search against a custom database containing all recognized and putative paramyxovirus species for which complete genomes are available. Based on these contigs (average coverage depth, 8×), primers were designed to determine the complete genome sequence by reverse transcriptase PCR (RT-PCR) and subsequent Sanger sequencing, and 5′ and 3′ rapid amplification of cDNA ends (RACE) using the 5′/3′ RACE kit (Roche) was used to complete the genome ends. Genome annotation was performed using the NCBI ORFfinder (www.ncbi.nlm.nih.gov/orffinder) and TMHMM Server v2.0 (www.cbs.dtu.dk/services/TMHMM) for transmembrane protein prediction. All tools were run with default parameters unless otherwise specified.

Based on its location (Rungwe District, Tanzania) and host of origin (*Lophuromys machangui*), this virus was given the name ruloma virus. Its genome consists of 20,454 nucleotides, in compliance with the “rule of six” for paramyxovirus genomes, and represents the largest mammalian paramyxovirus genome reported thus far (7). The genome has an average GC content of 33.24% and shares little similarity with known paramyxoviruses, as evaluated by pairwise sequence comparison (8) with Mount Mabu Lophuromys virus 1 and 2, showing the highest degree of nucleotide identity (both 41 to 42%). The genome structure, 3′-N-P/V/C-M-F-TM-G-L-5′, follows the typical paramyxovirus genome layout but includes an additional gene encoding a transmembrane protein between the F and G genes (9). Similar genome organizations are found only in members of the genus *Jeilongvirus*, which sometimes can even encode a second additional gene (SH) between this TM gene and the F gene. In accordance with its genome structure, phylogenetic analysis based on the protein sequence of all currently available (putative) orthoparamyxovirus species clusters ruloma virus as a sister lineage to all known members of the genus *Jeilongvirus* (Fig. 1). Interestingly, with a length of 784 amino acids, the G protein of ruloma virus is remarkably long, a trait thus far observed only in members of the “rodent clade” of the genus *Jeilongvirus* (5).

### Data availability.

The genome sequence of ruloma virus has been deposited in NCBI GenBank under the accession number MW579602. The Illumina data have been deposited in the NCBI SRA under the accession number SRR14154489.
